# Increasing motor cortex activation during grasping via novel robotic mirror hand therapy: a pilot fNIRS study

**DOI:** 10.1186/s12984-022-00988-7

**Published:** 2022-01-24

**Authors:** Dong Hyun Kim, Kun-Do Lee, Thomas C. Bulea, Hyung-Soon Park

**Affiliations:** 1 Department of Mechanical Engineering, Korea Advanced Institute of Science and Technology, Daejeon, 34141 South Korea; 2 Functional and Applied Biomechanics Section, Rehabilitation Medicine Department, Clinical Center, National Institutes of Health, Bethesda, MD 20892 USA

**Keywords:** Robotic mirror therapy, Stroke, Soft robotic glove, Functional near-infrared spectroscopy, Neurorehabilitation

## Abstract

**Background:**

Mirror therapy (MT) has been used for functional recovery of the affected hand by providing the mirrored image of the unaffected hand movement, which induces neural activation of the cortical hemisphere contralateral to the affected hand. Recently, many wearable robots assisting the movement of the hand have been developed, and several studies have proposed robotic mirror therapy (RMT) that uses a robot to provide mirrored movements of the unaffected hand to the affected hand with the robot controlled by measuring electromyography or posture of the unaffected hand. In some cases of RMT a mirror is placed to allow the person to observe only the unaffected hand but in others users simply observe the robotically assisted hand performing the mirrored movements, as was the case in this study. There have been limited evaluations of the cortical activity during RMT compared to MT and robotic therapy (RT) providing passive movements despite the difference in the modality of sensory feedback and the involvement of motor intention, respectively.

**Methods:**

This paper analyzes bilateral motor cortex activation in nine healthy subjects and five chronic stroke survivors during a pinching task performed in MT, RT, and RMT conditions using functional near infrared spectroscopy (fNIRS). In the MT condition, the person moved the unaffected hand and observed it in a mirror while the affected hand remained still. In RT condition passive movements were provided to the affected hand with a cable-driven soft robotic glove, while, in RMT condition, the posture of the unaffected hand was measured by a sensing glove and the soft robotic glove mirrored its movement on the affected hand.

**Results:**

For both groups, the RMT condition showed the greatest mean cortical activation on the motor cortex contralateral to the affected (non-dominant for the healthy group) hand compared to other conditions. Individual results indicate that RMT induces similar or greater neural activation on the motor cortex compared to MT and RT conditions. The interhemispheric activations of both groups were balanced in RMT condition. In MT condition, significantly greater activation was shown on the hemisphere ipsilateral to the affected (dominant for the healthy group) hand for both subject groups, while the contralateral side showed significantly greater activation for the healthy group in RT condition.

**Conclusion:**

The experimental results indicate that combining visual feedback, somatosensory feedback, and motor intention are important for greater stimulation on the contralateral motor cortex of the affected hand. RMT that includes these factors is hypothesized to achieve a more effective functional rehabilitation due to greater and more balanced cortical activation.

## Introduction

Individuals who experience stroke tend to lose motor function, and more than 70% of them have the upper limb affected. Particularly, hand function is most severely affected and also shows the worst response to standard of care therapy [1, 2]. Hand motor function can be improved by intense and repeated practice of functional movements through rehabilitation therapy. Repeated motor training is believed to improve motor functions because it induces neuroplastic changes that construct a new neural network in the intact cortical area, which replaces the function of the damaged area [3–5]. For effective rehabilitation, repeatedly providing neural stimulation around the motor and somatosensory cortex is important [6, 7]. A greater activation level of the cortical area near the motor cortex is observed after functional recovery likely indicating neuroplastic changes [8, 9].

Mirror therapy (MT) is a rehabilitation method of placing a mirror between the arms or legs so that the reflected movement of the non-affected limb gives an illusion of normal movement in the affected limb [10, 11]. MT is particularly used for the rehabilitation of individuals post-stroke who do not have the ability to conduct voluntary movements. The contralateral motor cortex of the affected limb is known to be stimulated by mirror therapy, although no voluntary movements are conducted [12–14]. However, the magnitude of the cortical activation is small compared to that of the unaffected limb as it does not convey actual movements and the corresponding somatosensory feedback.

Wearable robotic technologies enable assistance of limb movements for individuals with paralysis and other movement pathologies, and the use of wearable robots has expanded to rehabilitation therapy for various functional tasks. Recently, many researches have attempted to apply mirror therapy using wearable robots (robotic-mirror therapy, RMT) [15–17]. In RMT, the movement or muscle activation of the unaffected hand is measured and the wearable robot donned on the affected hand induces identical movement corresponding to the measurements. While some versions of RMT in the previous studies include a mirror to provide visual feedback of the mirrored less affected hand, other RMT systems do not. In this latter case, the term “mirror” indicates that the robotic assistance provided to the more affected hand is “mirroring” the movement of less affected hand. Unlike MT that only provides visual feedback, RMT provides both visual feedback and somatosensory feedback by providing passive movements. However, the neural effect of the RMT compared to MT is still a question and needs to be studied to understand the effect on actual functional recovery.

There are various methods available to observe neural activation of the brain during functional movements. Functional near-infrared spectroscopy (fNIRS) is one of these methods, which non-invasively measures brain activation by analyzing the hemodynamics of the cerebral vessels through near-infrared light. Movement-related cortical activity, including both the area and magnitude of activations, can be quantified with fNIRS by placing multiple light-emitters and detectors on the scalp around the motor cortex. Previous studies have evaluated the effectiveness of robotic and/or sensorized gloves using fNIRS and observed increased cortical activation [18, 19]. By spatial analysis of fNIRS previous studies have also identified the correlation between the variation of functional recovery of MT among subjects and the shift of cortical activation on the precuneus region [20], and analyzed the functional laterality according to the time after a stroke [21].

In this paper, we analyzed the neural effect of MT, robotic therapy (RT), and RMT in repetitive pinching movements. RT was tested, in addition, to observe the neural effect when no movement intention was involved. The RMT was conducted by measuring the movement of the unaffected hand with a custom-designed sensor glove and inducing movement of the affected hand with a soft robotic glove that could assist 4-DOF movements [22] previously developed from our research group. RT was conducted by moving the affected hand with the soft robotic glove without involving movement of the unaffected hand. fNIRS was used to measure neural activity in the brain during MT, RT, and RMT. Neural effects of each condition on healthy subjects and stroke survivors were analyzed and compared.

## Methods and materials

### A. Participants

Nine healthy subjects (43.8 ± 14.3 yrs) and five stroke survivors with hemiplegia (60.6 ± 6.2 yrs) participated in the experiment and the demography is shown in Table 1. The experimental protocols were approved by the Institutional Review Board at the Korea Advanced Institute of Science and Technology (KH2018-127); written informed consent was obtained from each subject before participation.

**Table 1:** Subject demographics

Subject	No	Sex	Age (yrs)	Time since stroke (months)	Affected (Stroke) or dominant (healthy) side	Brunnstrom stage
Stroke	P1	M	60	336	Right	4
	P2	M	59	84	Right	4
	P3	F	52	84	Right	4
	P4*	F	69	372	Left	2
	P5	M	63	132	Left	3
Healthy	S1	F	23	–	Right	–
	S2	M	30	–	Right	–
	S3	M	24	–	Right	–
	S4	M	51	–	Right	–
	S5*	F	49	–	Right	–
	S6	F	50	–	Right	–
	S7	M	62	–	Right	–
	S8	F	49	–	Right	–
	S9	F	56	–	Right	–

*Excluded subjects with no significant fNIRS signal with respect to motion

### B. Experimental apparatus and setup

#### Soft robotic glove

**Fig. 1:**
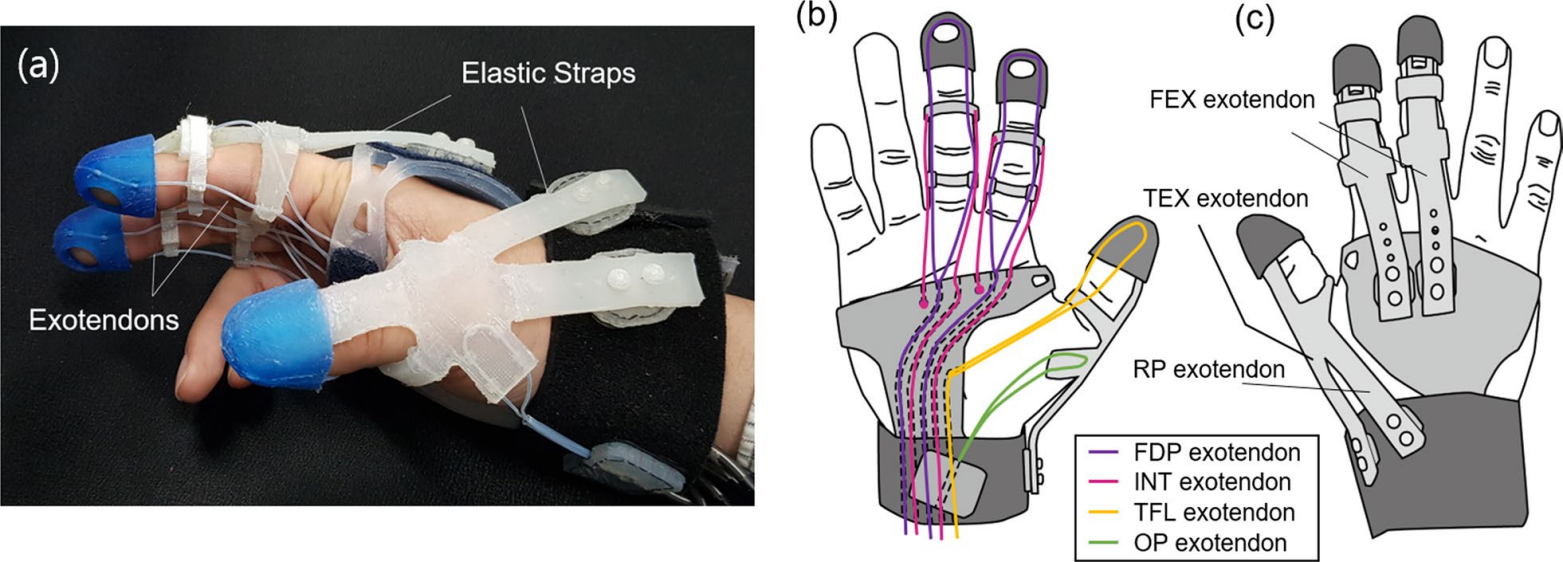
Design overview of the soft robotic glove. **a** Overview of the soft robotic glove, **b** routing of active exotendons, **c** routing of passive exotendons

A 4-DOF soft robotic glove [22] was used to assist the movements of the affected hand of the stroke survivors and non-dominant hand of the healthy subjects during the therapy. The soft robotic glove is actuated by elastic straps (passive exotendons) and cables (active exotendons) that replicate the orientation of the hand’s musculotendinous units (Fig. 1a).

The passive exotendons keep the fingers and thumb extended by elastic force and they are routed as in Fig. 1c. The finger-extensor (FEX) exotendon and thumb-extensor (TEX) passed the dorsal aspect of the finger and thumb, respectively, while the reposition (RP) exotendon inserts to the TEX exotendon near the dorsal aspect of the metacarpophalangeal (MCP) joint and passes the dorsal aspect of the wrist. FEX and TEX exotendons induce extension of the finger and thumb, respectively, while the RP exotendon induces reposition (combined movement of extension and adduction) of the carpometacarpal (CMC) joint. The assisted force could be adjusted by changing the stretched length of the passive exotendon with a hook-and-loop fastener button.

The active exotendons consisting of cables are connected with servomotors that actively provide force to the exotendon. The active exotendons are routed as in Fig. 1. The flexor digitorum profundus (FDP) exotendon (Fig. 1b, purple line) passes the palmar aspect of the finger joints, while the intrinsic (INT) exotendon (Fig. 1b, red line) is inserted at dorsal aspect of the FEX exotendon near the middle phalanx and passes the dorsal aspect of the proximal-interphalangeal (PIP) joint, lateral side of the proximal phalanx, and palmar aspect of the MCP joint. The FDP exotendon flexes all finger joints while the INT exotendon flexes the MCP joint and extends the distal-interphalangeal (DIP) and PIP joints. The movement of PIP and MCP joints of the finger could be controlled separately by controlling FDP and INT exotendons. The opposition (OP) exotendon (Fig. 1b, green line) is inserted on the proximal aspect of the first metacarpal bone and passes the palmar aspect of the wrist, which induces opposition (combined movement of flexion and abduction) of the CMC joint of the thumb. The thumb-flexor (TFL) exotendon (Fig. 1b, yellow line) passes the dorsal aspect of the thumb joint and induces flexion of all joints.

In total, three digits are actuated with the soft robotic glove including the index finger, middle finger, and thumb. The active exotendons of the index and middle finger are connected to the same motor and actuated together, while the thumb is actuated separately.

#### Sensor glove

**Fig. 2:**
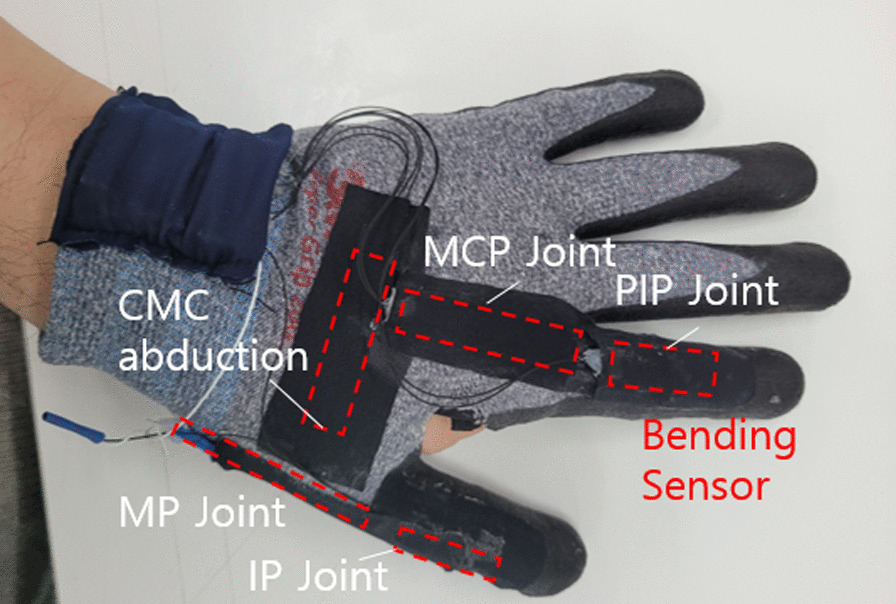
Overview of the sensor glove and location of bending sensors

The sensor glove embeds bending sensors (Bend sensor, Flexpoint Sensor Systems Inc., Draper, UT, USA) that changes resistance depending on the amount of bending. The bending sensors are placed on the dorsal aspect of joints to measure joint angles of the finger and thumb. The sensor glove measures the flexion angle of the PIP and MCP joint of the index finger, combined flexion of the interphalangeal (IP) joint and MCP joint of the thumb, and opposition of the thumb CMC joint. The location of the sensors for measuring the joint angles are shown in Fig. 2.

#### Control strategy

The soft robotic glove is controlled to generate the same hand posture measured (joint angle measurements) from the sensor glove (${{q}}_{{d}}$). The length of the active exotendons (${l}$) are controlled to the length that corresponds to the measured posture (${{l}}_{{d}}$). The length of the active exotendon corresponding to the joint angle vector ${q}$ is given as

1 $${{l}}_{{d}}={{T}}_{{a}}^{{T}}\left({{q}}_{{d}}\right){{q}}_{{d}}$$

where, ${{T}}_{{a}}^{{T}}$ represents the exotendon force-to-torque transformation matrix. The elements of the transformation matrix are instantaneous moment arms of the corresponding exotendon spanning the index finger, middle finger, and thumb joints.

An impedance controller was used for controlling the active exotendon as follows:

2 $${{f}}_{{a}}=-{{K}}_{{l}}\left({l}-{{l}}_{{d}}\right)-{{D}}_{{l}}\left(\dot{{l}}-{\dot{{l}}}_{{d}}\right)$$

where, ${{f}}_{{a}}$ represents the tensional force of the active exotendon, ${{K}}_{{l}}$ and ${{D}}_{{l}}$ represent the proportional and derivative gain matrix, respectively. By substituting (1) into (2) and letting ${\dot{{l}}}_{{d}}$ = 0, ${{f}}_{{a}}$ becomes

3 $${{f}}_{{a}}=-{{K}}_{{l}}\left({{T}}_{{a}}^{{T}}\left({q}\right){q}-{{T}}_{{a}}^{{T}}\left({q}\right){{q}}_{{d}}\right)-{{D}}_{{l}}{{T}}_{{a}}^{{T}}\left({q}\right)\dot{{q}}$$

The effective stiffness of joints could be adjusted with the feedback control proportional gain matrix for exotendon length (${{K}}_{{l}}$). For biomechanically intuitive adjustments, a diagonal matrix was used for ${{K}}_{{l}}$ as it enables adjustment of the stiffness of each exotendon. The adjustability of effective stiffness of joints enables the robotic glove to provide the same posture regardless of joint stiffness that varies between subjects [23].

#### Experimental setup

The soft robotic glove and the sensor glove was donned on the affected hand and the unaffected hand, respectively, for stroke subjects, whereas they were donned on the dominant hand and non-dominant hand, respectively, for healthy subjects. The subjects sat on a chair and placed their hand on the table.

**Fig. 3:**
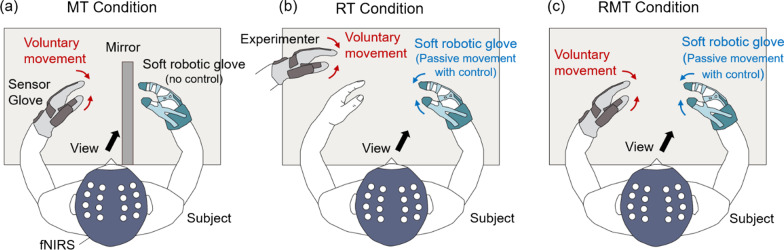
Experimental setup of each test condition: **a** MT condition, **b** RT condition, **c** RMT condition

In the MT condition, a 26 cm × 40 cm mirror was placed in front of the body on the desk in the position shown in Fig. 3a. The mirror was vertical to the plane of the desk and directed to the hand with the sensor glove. The orientation of the mirror was adjusted until the subject feels the reflected image of the unaffected (or non-dominant) hand as his/her affected (or dominant) hand. The experiments in RT and RMT condition were conducted without the mirror. The stiffness of the exotendon was tuned to each individual so that the subject could closely follow the pinching motion conducted by the hand with the sensor glove.

**Fig. 4:**
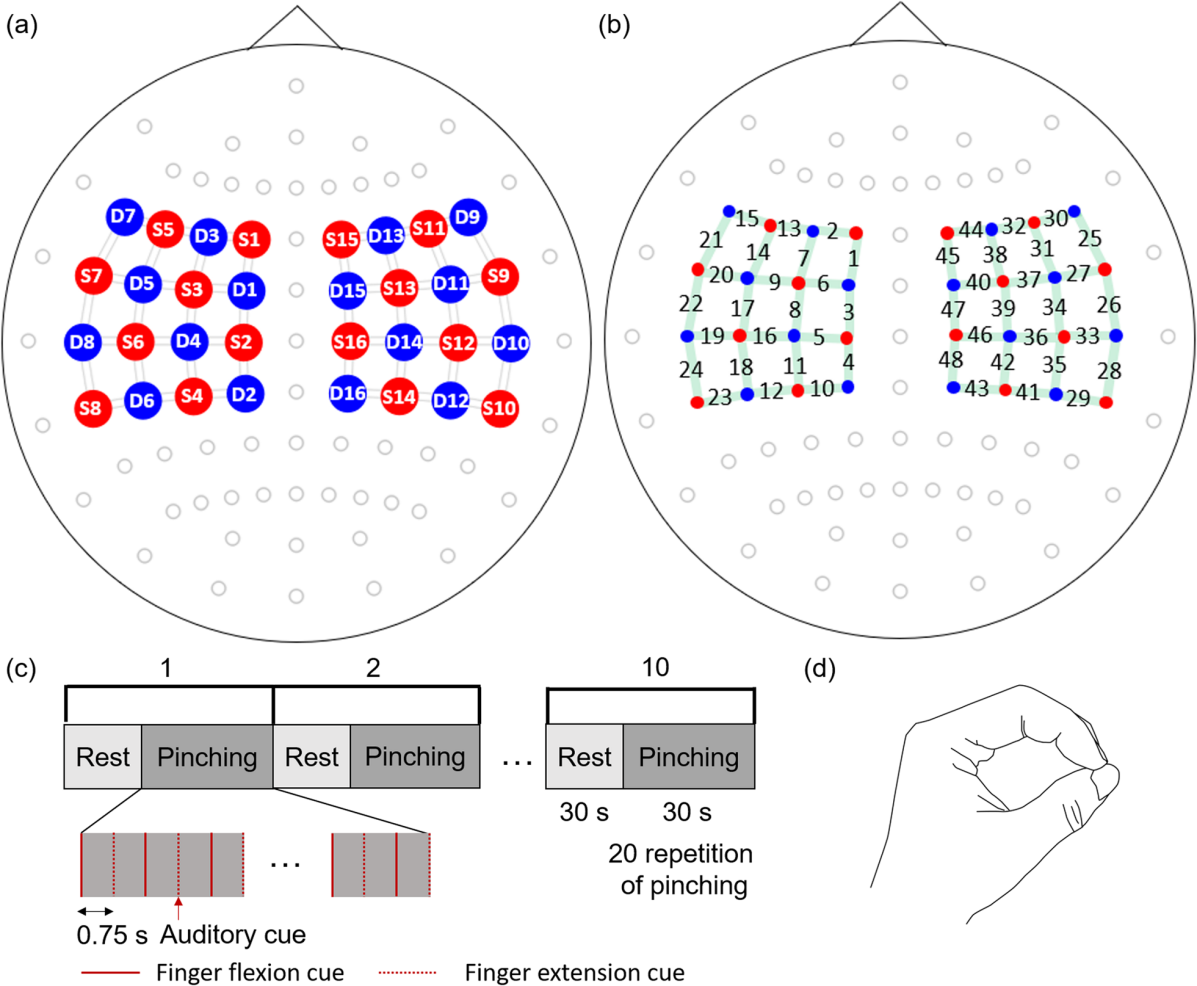
Overview of experimental task and fNIRS setup: **a** Motor cortex montage of 16 sources (S1 ~ S16, red dots) and 16 detectors (D1 ~ D16, blue dots). **b** channel locations (1–48). **c** Schematic diagram of the experiment for each task condition. **d** Target pose of pinching to be generated by the soft robotic glove

NIRSport 2 (fNIRS, NIRx Medical Technologies, Glen Head, NY, USA) was used to measure brain activation. A 16 × 16 motor cortex montage (Fig. 4a) was adopted to measure the brain activation related to the tasks. Sources marked as red dots in the montage produce lights at two wavelengths (760 nm and 850 nm) that can penetrate through the skull and be absorbed by hemoglobin in the cerebral cortex. With data collected at each wavelength and using Beer-Lambert law, detectors marked as blue dots measure the level of oxygenated hemoglobin (HbO, 850 nm) and deoxygenated hemoglobin (HBR, 760 nm) with sampling rate 4.4 Hz [24]. Each pair of source and detector is marked as a green link in Fig. 4b, and the distance between them were kept to be 3 cm. The source and detector construct 48 channels in total. The channel location and its numbering are marked in Fig. 4b.

### C. Experimental procedure

The experiments in each condition were arranged in a block design paradigm (Fig. 4c). Each 30 s block consisted of 20 pinching trials. An auditory cue was provided at 0.75 s intervals (1.33 Hz) during the pinching block trial to indicate timing to open and close the hand. Each pinching block was followed by a resting block lasting 30 s. Pinching and resting blocks were repeated 10 times for each condition. Pinching was conducted by using the tip of the index finger, middle finger, and thumb (Fig. 4d). In the resting state, subjects were asked to relax. During the pinching task, the subjects were asked to observe the movement of the hand wearing the soft robotic glove in the RT and RMT condition, and to observe the mirror image of the unaffected (or non-dominant) hand under MT condition. The details of the movement on each condition are as follows.

#### MT condition

Subjects were asked to repeat pinching motion for the hand wearing the sensor glove according to the auditory cue. Subjects were asked not to voluntarily move the hand wearing the actuating glove. The actuating glove did not assist hand movement in this condition. Subjects were instructed to visually observe the pinching hand in the mirror.

#### RT condition

Subjects were asked not to voluntarily move both hands. The experimenter wore the sensor glove and repeated pinching motion according to the auditory feedback. Pinching movements were performed by controlling the soft robotic glove according to the hand posture measurements of the sensor glove. As the soft robotic glove is controlled by the experimenter, this condition does not include the subject’s intention to move. Subjects were instructed to visually observe the hand being moved by the actuated glove.

#### RMT condition

Subjects were asked not to move the hand wearing the soft robotic glove, and to repeat pinching motion for the hand wearing the sensor glove according to the auditory cue. The soft robotic glove was controlled to perform pinching movement according to the hand posture measurements of the sensor glove. As the soft robotic glove is controlled by the subject, this condition includes the subject’s intention to move. Subjects were instructed to visually observe the hand being moved by the actuated glove.

**Fig. 5:**
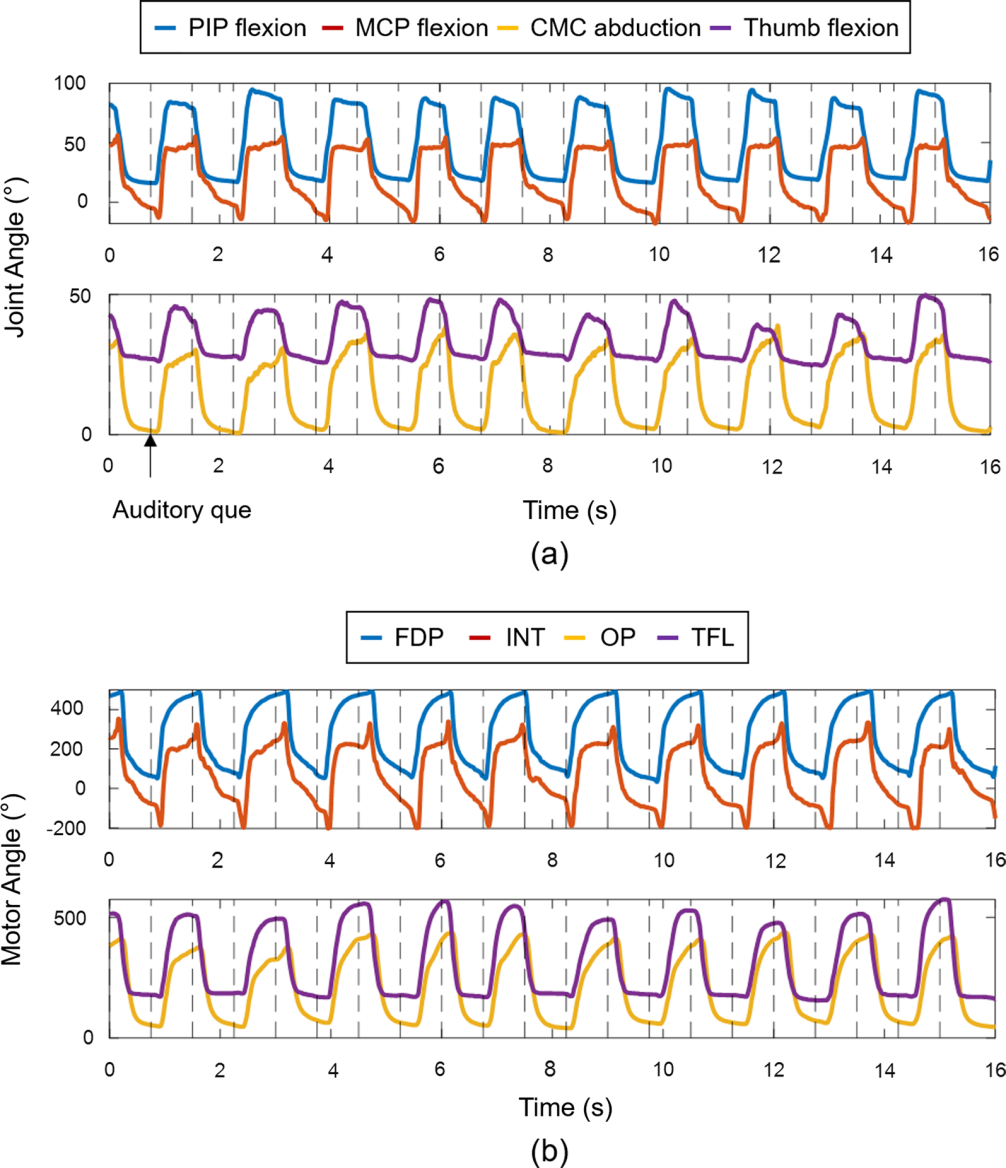
Joint angle measured by the sensor glove and angular displacement of the motor actuating the soft robotic glove during the experiment in RMT condition (subject P1): **a** Joint angle measured by the sensor glove, **b** angular displacement of the motors that actuate the active exotendons. The vertical dashed lines represent the timing of the auditory que

The representative results showing the sensor glove measurements and angular displacement of the motors actuating the active exotendons for a single participant post-stroke is illustrated in Fig. 5. Note that the participant was able to move their unaffected hand according to the auditory cue and the robotic glove was actuated based on the sensor glove measurements (Fig. 5).

### D. Data analysis

Data analysis was composed of three steps. First, ‘Data Filtering’ was applied to extract frequency components that are related to hemodynamic signal. Second, in ‘Significant Channel Selection’, we selected the channels that are significantly activated by observing the change of HbO and HbR during the task period and using functional connectivity (FC) analysis, which analyzed the correlation coefficient of signals between channels to identify functionally related brain regions [25, 26]. To compare the effect of task conditions on the brain, in ‘Deriving Quantitative Indices’, we introduce indices that quantify the activation level of each channel and interhemispheric balance between channels symmetrically located in the ipsilateral and contralateral hemispheres around the motor cortex. Throughout the data analysis and experimental results, the ‘contralateral side’ denotes the brain hemisphere opposite of the hand wearing the soft robotic glove, which is the affected hand for the stroke group and non-dominant hand for the healthy group.

#### 1) Data filtering

We considered HbO signal as a primary indicator for brain activation as it is more sensitive to the change of blood flow than HbR [27]–[29]. We filtered the HbO and HBR signal with a band-pass filter with cut-off frequencies at 0.01 Hz and 0.09 Hz to remove high-frequency signals such as heartbeat and respiration, and low-frequency components caused by the change of scalp condition over time [30, 31].

#### 2) Significant channel selection

Channels that satisfied the following two conditions were identified as having significant task-related activity. First, each channel that showed a significant change in HbO and HbR signals during task periods were selected. By conducting t-test as shown in Eq. (4) (${c}$: channel selection vector, ${G}$: design matrix, ${\widehat{\sigma }}^{2}:$ residual sum-of-squares devided by the degrees of freedom), the channels that rejected the null hypothesis $\beta =0$ (p < 0.05) were considered to be significant [32].

4 $$\mathrm{t}= \frac{{{c}}^{T}\widehat{{\varvec{\upbeta}}}}{\sqrt{{\widehat{\sigma }}^{2}{{c}}^{T}{({{G}}^{T}{G})}^{-1}{c}}}$$

**Fig. 6:**
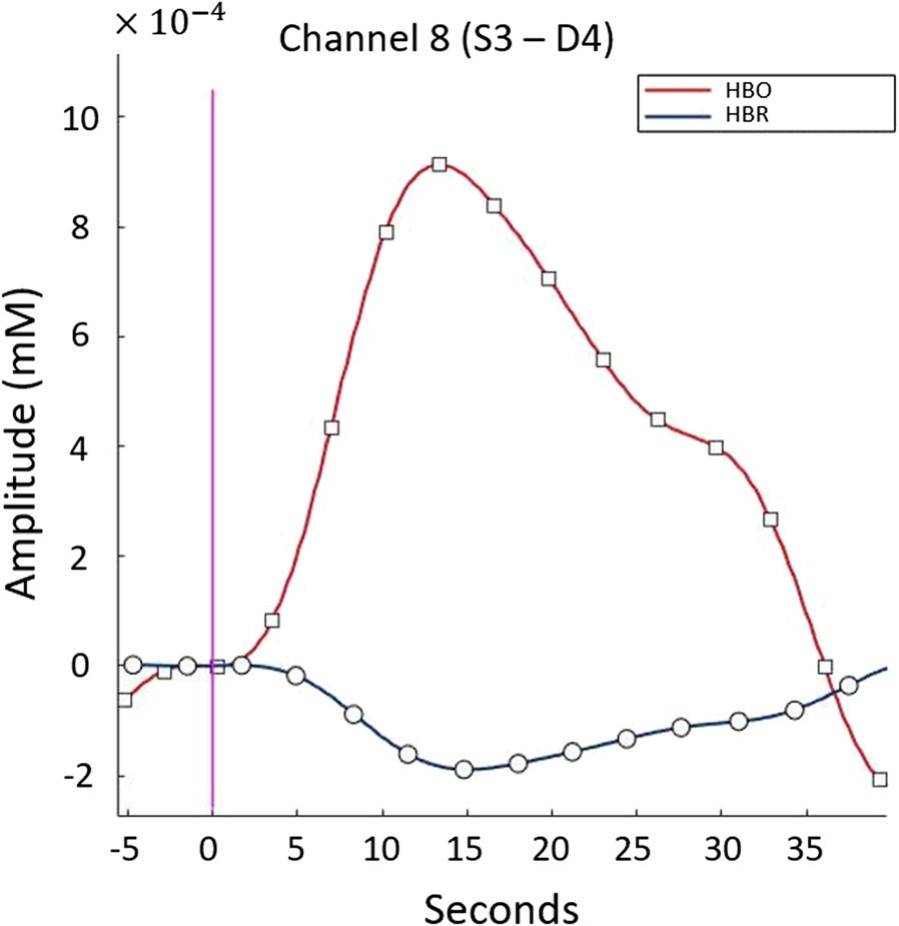
Block averaged HBO and HBR plot of channel 8 of patient 1 RMT. Increase in HBO and decrease in HBR can be observed

The detailed description for the parameters is in the equations of the following section. A representative example of the change of HbO and HbR over time is shown in Fig. 6. Second, we calculated the Pearson correlation of HbO and HbR time series data between channels to identify and retain task related channels and reject channels that are highly affected by external noise. This type of FC analysis has previously been shown to identify functionally related brain regions [25, 26]. Channels in the same hemisphere with HbO and HbR that show higher correlation than 0.8 with at least one other channel were retained. The significant channels satisfying these two requirements have considerable activation and a high correlation with other channels, so they can be considered to detect task-related hemodynamic activity.

#### 3) Derivation of Quantitative Index

##### a. Activation level (general linear model)

**Fig. 7:**
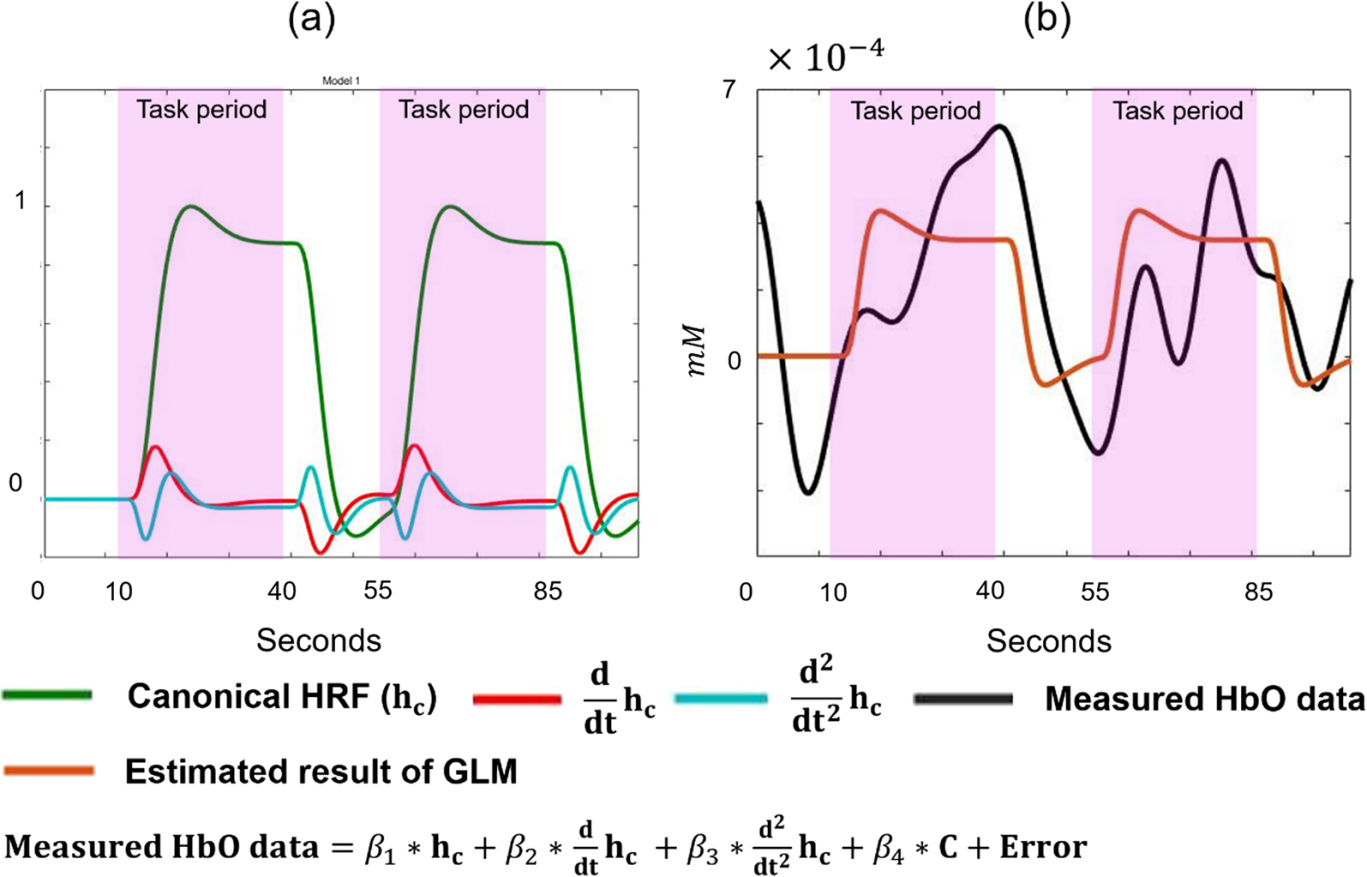
Schematic description of GLM method: **a** Time series model of canonical HRF (first column of design matrix), temporal derivative of HRF (second column of design matrix), and dispersion derivative of HRF (third column of design matrix). **b** Measured HbO data and fitted model which is ${G}\widehat{{\varvec{\upbeta}}}$

To estimate the activation level of each significant channel, we adopted the general linear model (GLM) [33]. Using the least square method, GLM method fits series of hemodynamic response functions (HRFs) to pre-processed time series data of each channel, and estimates scaling coefficients ${\varvec{\upbeta}}$ values. Note that HRF is a functional modeling of hemodynamic change in response to neural activation, and among various models we used canonical HRF model [34]. Equation (5)-(8) and Fig. 7 describe GLM method.

5 $${Y}= \left[{{h}}_{{c}},\frac{{d}}{{d}{t}}{{h}}_{{c}},\frac{{{d}}^{2}}{{{d}{t}}^{2}}{{h}}_{{c}},{C}\right]\bullet {\varvec{\upbeta}}+{E}$$

6 $${Y}={G}{\varvec{\upbeta}}+{E}$$

7 $$\widehat{{\varvec{\upbeta}}}={({{G}}^{{T}}{G})}^{-1}{{G}}^{{T}}{Y}$$

8 $$\widehat{{\varvec{\upbeta}}}={\left[{\beta }_{1}, {\beta }_{2}, {\beta }_{3}, {\beta }_{4}\right]}^{{T}}$$

As shown in Eqs. (5) and (6), we composed design matrix ${G} \left({{R}}^{n\times m}\right) (m=4)$ with canonical HRF (${{h}}_{{c}}$), temporal derivative of HRF ($\frac{{d}}{{d}{t}}{{h}}_{{c}}$), dispersion derivative of HRF ($\frac{{{d}}^{2}}{{{d}{t}}^{2}}{{h}}_{{c}}$) and constant term **C**. ${Y} \left({{R}}^{n}\right)$ is pre-processed time series data of a channel and ${E} \left({{R}}^{n}\right)$ is the error term to minimize.

With least square method in Eq. (7), $\widehat{{\varvec{\upbeta}}} \left({{R}}^{m}\right)$ can be obtained. Among the elements in $\widehat{{\varvec{\upbeta}}}$, we considered ${\beta }_{1}$ as the primary indicator of signal magnitude for each channel, and compared it between task conditions and subjects. For group-level analysis, we averaged ${\beta }_{1}$ of each channel over the subjects in each group. ${\beta }_{1}$ of not significantly activated channels was set to be 0.

We utilized a linear mixed effects model to investigate the influence of task condition, group and their interaction on activation magnitude considering difference between participants as random effect. The formula for model specification was ‘beta ~ group + condition + group*condition + (1|paticipants)’.

##### b. Laterality

We also compared the interhemispheric balance of brain activation for each task condition and subject group. For this purpose, we defined laterality $L$ as follows [35]:

9 $$L=\frac{{\beta }_{1, ipsi}-{\beta }_{1,contra}}{{\beta }_{1, ipsi}+{\beta }_{1, contra}}$$

where, ${\beta }_{1, ipsi}$ and ${\beta }_{1, contra}$ are ${\beta }_{1}$ values of ipsilateral and contralateral side of paired channels.

We paired channels in symmetric position on the ipsilateral and contralateral hemisphere. *L* value is between −1 and 1. When it is negative, activation on contralateral side is dominant, and when it is positive, ipsilateral side is dominant. When *L* is close to zero, it indicates that the activation level is symmetric and balanced. We obtained laterality of channel pairs neighboring to C1, C2, C3 and C4 position of 10–20 international EEG system that are known to be primary motor cortex area [36]. The significance of laterality was determined by a linear mixed effects models and its formula was ‘laterality ~ side + (1|participants)’ for each channel pair.

## Results

For channel-wise comparison, the data was flipped across the midline in the participants who wore the soft robotic glove on left hand such that data from channel 1 to 24 (left-side channels in Fig. 4) are represented as being contralateral to the robotic glove and 25 to 48 (right-side channels in Fig. 4) are represented as ipsilateral to the robotic glove for all participants.

**Fig. 8:**
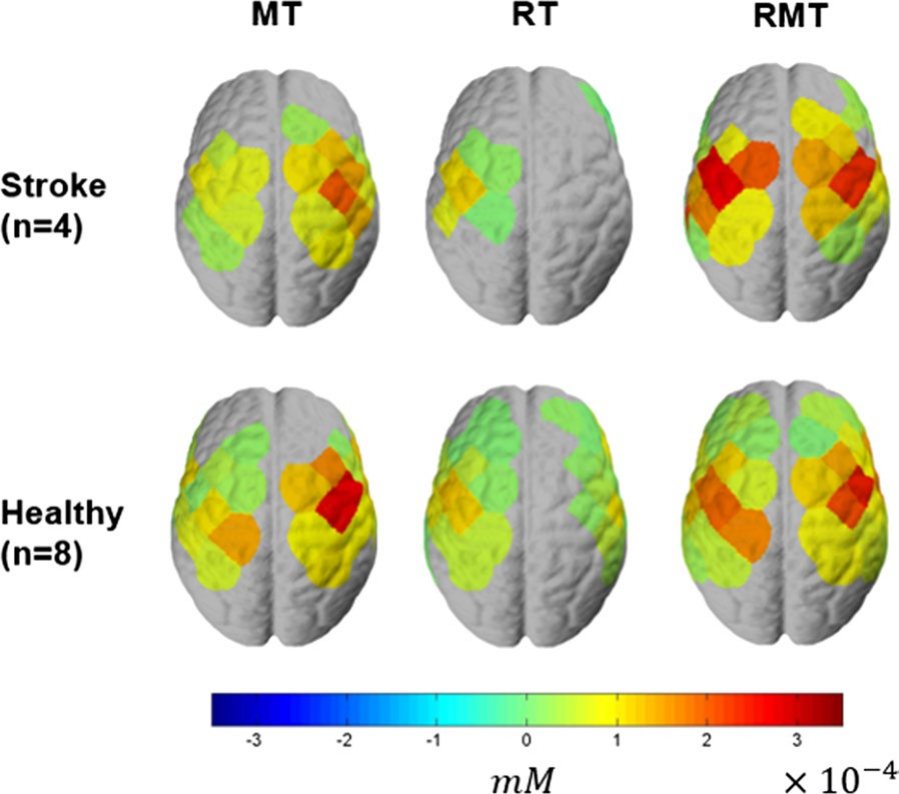
Group-level cortical activation (${\beta }_{1})$ of stroke participants (n = 4) and healthy subjects (n = 8): The left hemisphere, in the figure, represents the contralateral side of the affected hand for the stroke group and non-dominant hand for the healthy group

**Fig. 9:**
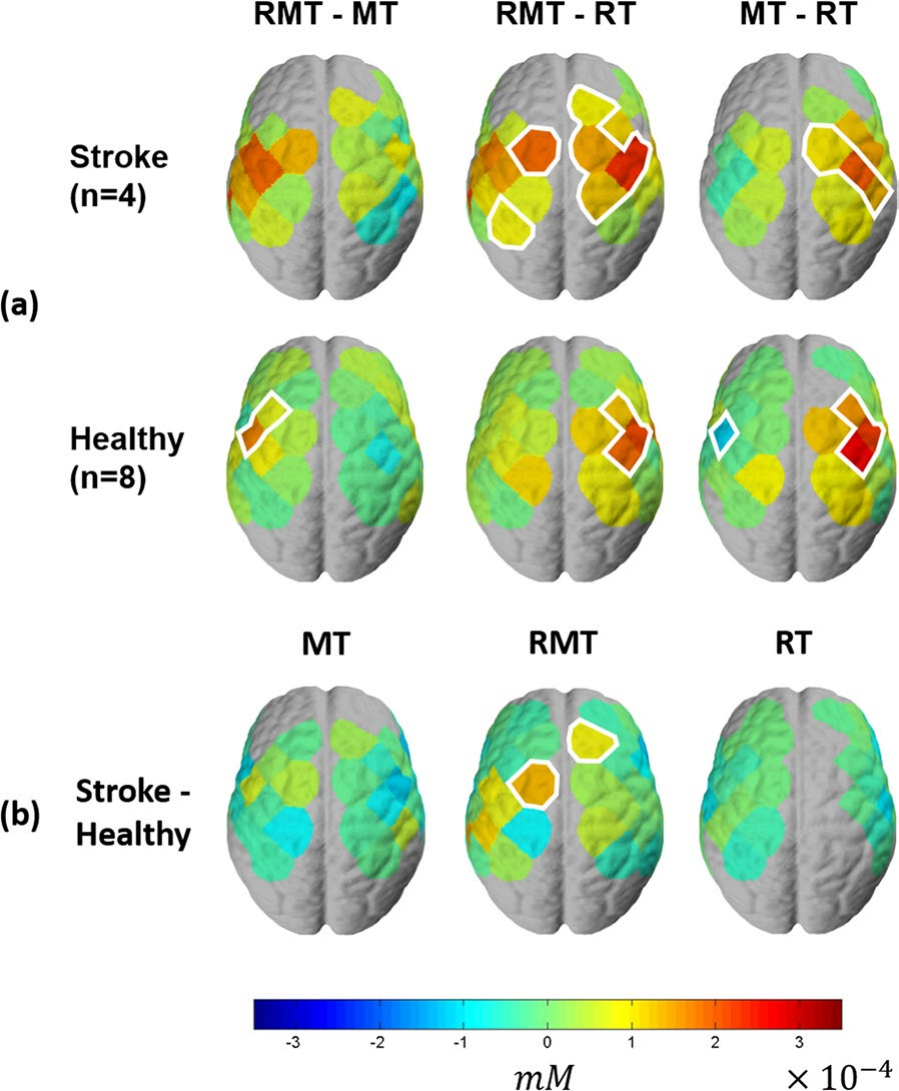
Group-level contrast of cortical activation: **a** Group-level contrast between training conditions (MT vs. RMT vs. RT) for each group, **b** Group-level contrast between subject groups (stroke vs. healthy). T-test based on mixed effects model was performed, and the threshold of significance was set to be p < 0.05. The significant channels are highlighted by white silhouette

**Fig. 10:**
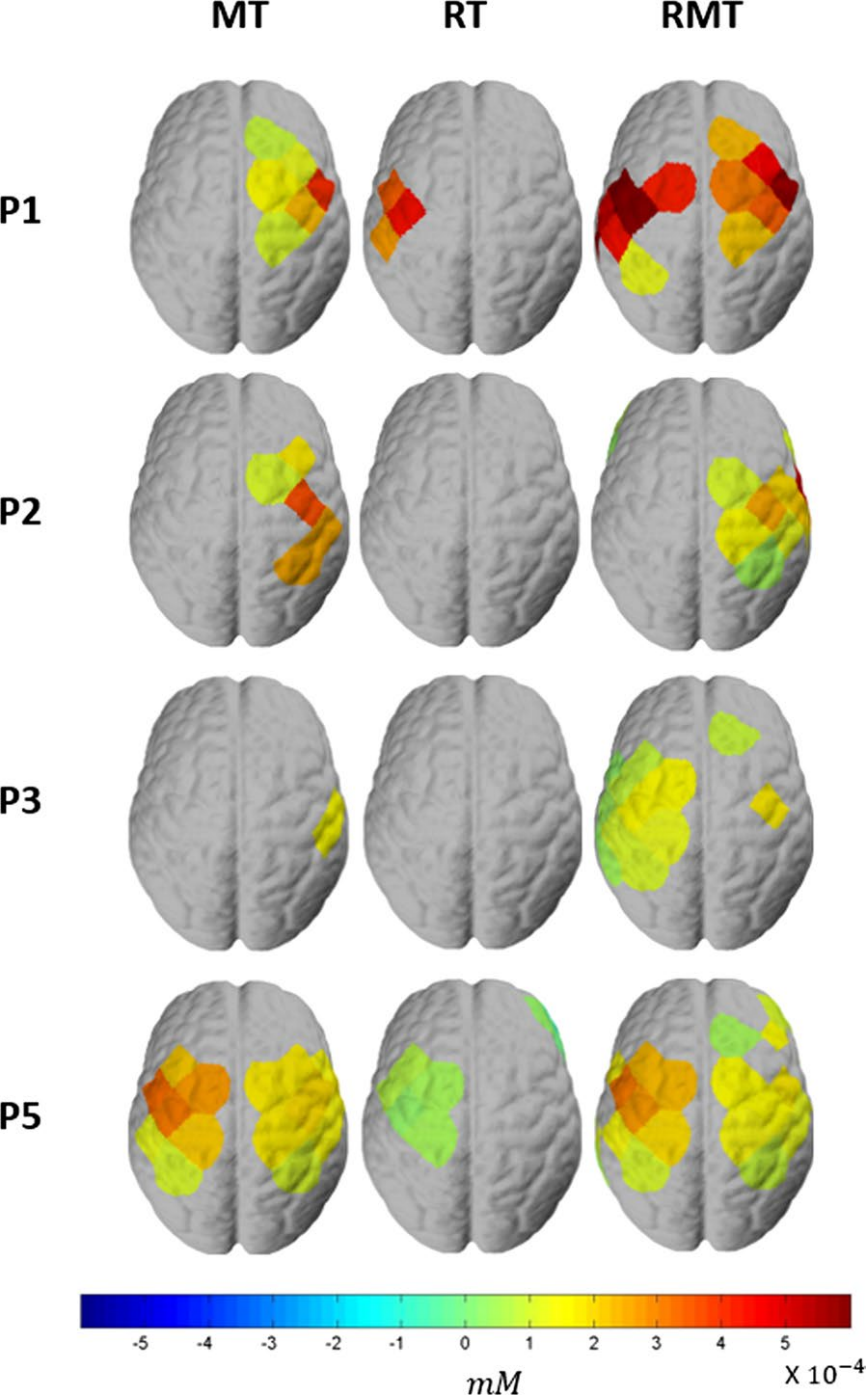
Neural activation level (${\beta }_{1}$) of each patient: The left hemisphere, in the figure, represents the contralateral side of the affected hand for the stroke group and non-dominant hand for the healthy group

1. **Activation Level (HbO concentration)**
  For both stroke and healthy groups, MT activated both sides of the motor cortex, but greater activation was observed on the ipsilateral hemisphere to the robotic glove, which was contralateral to the hand that performed the movement (Fig. 8). RT primarily activated the motor cortex in the hemisphere contralateral to the robotic glove, while RMT induced activation of both sides (Fig. 8) with similar magnitude. RMT induced the strongest activation on contralateral channels (Fig. 8). Particularly, on contralateral side of RMT, channel 5 and 8 showed the highest activation (Fig. 8), which are located around C4 (or C3) that is known to be the area responsible for hand movement [36]. MT induced activation on both sides, but stronger activation on ipsilateral hemisphere to the robotic glove, which is contralateral to the hand executing the pinch movements. RT showed primary activation on the contralateral hemisphere but its magnitude was smaller than in RMT.
  The group-level contrast is shown in Fig. 9. Significantly greater activation on the contralateral hemisphere was shown for stroke survivors in RMT condition compared to RT condition even though both conditions involved the robotic glove moving the hand. Specifically, RMT induced greater activation on the contralateral primary motor cortex (channel 3) and contralateral somatosensory cortex (channel 10) compared to RT (Stroke RMT-RT, Fig. 9a). This effect was not observed in the healthy group. As expected due to the presence of contralateral hand moment, greater motor cortex activation was observed in both stroke and healthy groups on the hemisphere ipsilateral to the robotic glove in RMT compared to RT. The healthy group showed significantly greater activation on the contralateral primary motor cortex in RMT condition compared to MT condition (channels 6, 8, and 18; Fig. 9a) whereas contralateral activation magnitude was greater RMT compared to MT in stroke group but it did not reach significance at the group level (0.1 < p < 0.05). MT induced significantly smaller activation on contralateral primary motor cortex (channel 8) compared to RT in healthy group, while no significant differences were shown in the cortical activation in the contralateral hemisphere between MT and RT in the stroke group. For the ipsilateral hemisphere, MT showed significantly larger activation than RT because of the voluntary movement of the contralateral hand (unaffected hand for stroke subjects and nondominant hand for healthy subjects).
  The cortical activation showed significant differences between subject groups (stroke vs. healthy) in RMT condition on the ipsilateral premotor cortex (channel 45) and contralateral primary motor cortex (channel 3 and 18), while no significant difference was observed between subject group in RT and MT condition (Fig. 9b).
  Additional analysis was conducted at the individual level in participants post-stroke to see how training conditions affect the brain (Fig. 10) for each individual. P4 was excluded because there were no channels that satisfy the significant channel selection conditions. Only one participant (P5) had significant activation the contralateral hemisphere to the affected hand during MT whereas all four showed significant activation on the ipsilateral hemisphere that was contralateral to the hand being moved. P1, P3 and P5 showed significant contralateral hemisphere activation during RMT whereas P2 retained a similar pattern as MT with only significant activation on the ipsilateral hemisphere to the robotic glove for RMT. P5 showed almost the same activation on the contralateral side for MT and RMT. For RT, P2 and P3 showed no significant activation on both contralateral and ipsilateral hemispheres. P1 and P5 had activated channels on the contralateral hemisphere for RT, but the magnitude and number of activated channels were less than in RMT.
2. **Laterality**

**Fig. 11:**
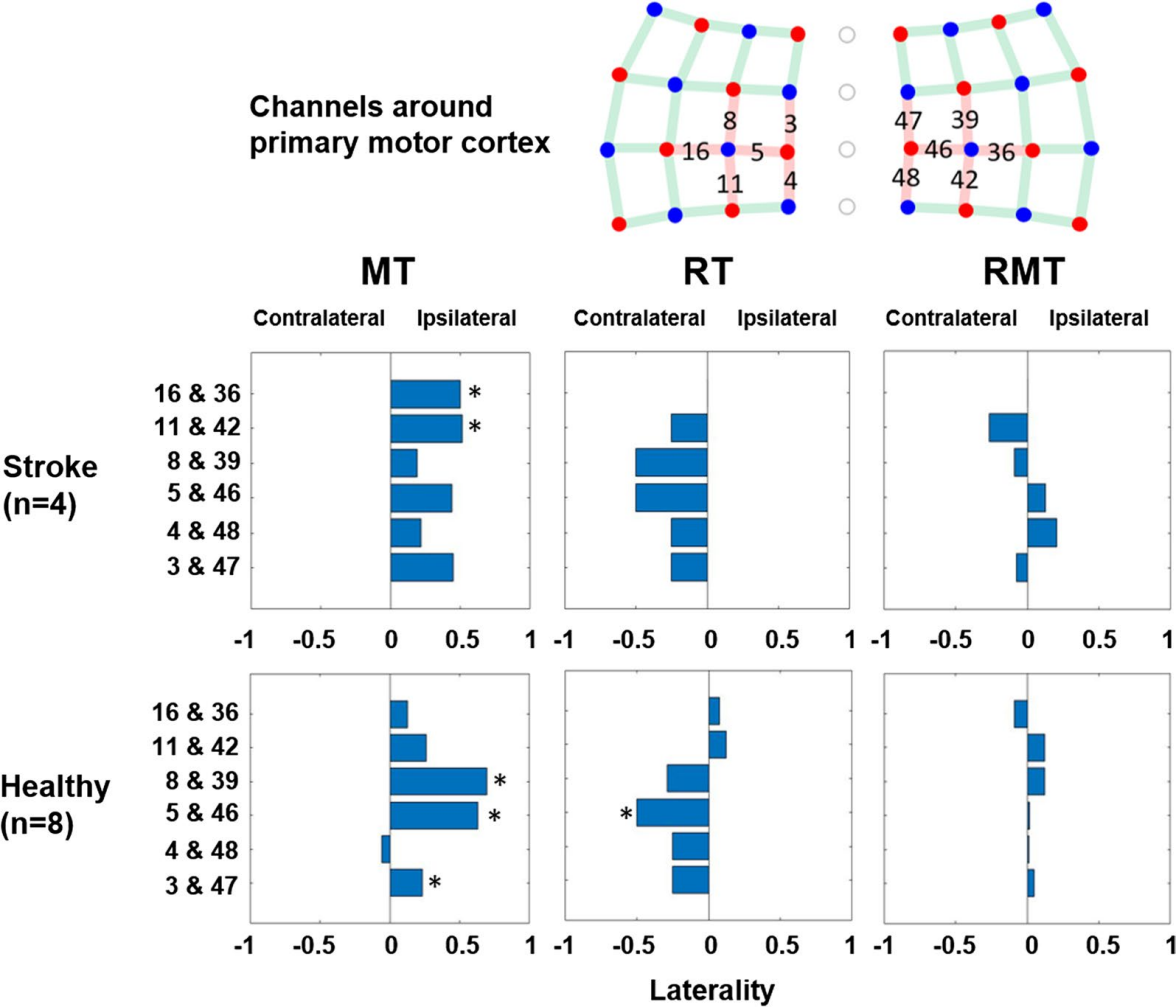
Laterality result for subject groups and task conditions: MT and RT result respectively show ipsilateral and contralateral dominant result, but RMT shows balanced activation between ipsilateral and contralateral side of the brain. The contralateral side denotes the hemisphere contralateral to the affected hand for the stroke group and the non-dominant hand for the healthy group. It represents the opposite side of the hand with the soft robotic glove. The ipsilateral side represents the hemisphere ipsilateral to the affected and non-dominant hand. (Asterisk (*): significant bias with p < 0.05)

For MT condition, the laterality was biased toward the hemisphere contralateral to the hand performing the movement (Fig. 11). The stroke group showed significant bias to the hemisphere ipsilateral to the robotic glove in 11 & 42 and 16 & 36 channel-pairs, while, in the healthy group, 3 & 47, 5 & 46 and 8 & 39 showed significant bias to the same hemisphere. Although not significant, most of the channel-pairs (except 4 & 48 of the healthy group) in both subject groups resulted in positive laterality which is bias to the ipsilateral hemisphere to the robotic glove, which was not moving in MT. For RT condition, there was no significant bias in the stroke group, while 5 & 46 channel-pair showed significant bias to contralateral hemisphere to the robotic glove in the healthy group. Most of the channel-pairs (except 11 & 42 and 16 & 36 of healthy group) in both groups showed negative bias which is the bias to the contralateral hemisphere. Lastly, there was no significantly biased channel pair in RMT in either group (Fig. 11) demonstrating balanced cortical activation.

## Discussion

In this study, we analyzed the neural effect of MT, RT, and RMT of the hand from the cortical activation measured by fNIRS. RMT was able to be conducted with the proposed hand rehabilitation system that assists the 4-DOF movement of the affected hand (dominant hand for healthy subjects) with the soft robotic glove according to the movement of the unaffected hand (non-dominant hand for healthy subjects) measured with the sensor glove. RMT induced larger activation on the contralateral motor cortex of the affected hand compared to MT and RT. By comparison between subject groups, significantly greater activation was observed in the primary motor cortex on the contralateral side of stroke subjects compared to healthy only for RMT condition, which may indicate that combining motor intension, visual feedback, and somatosensory feedback is important for inducing greater activation in the motor cortex following a stroke.

Providing sensory feedback with proper modality is important for greater activation of the primary motor cortex, which may promote functional recovery. MT provides a visual illusion of the movement of the affected hand through the reflected image of the unaffected hand, while RMT directly provides visual and somatosensory feedback of the affected hand. Higher activation was observed on the contralateral motor cortex in the condition with both visual feedback and somatosensory feedback (RMT, Fig. 8) compared to the condition with visual illusion (MT). We should note that between-subject variations were observed in activation of the contralateral motor cortex. Still, RMT condition showed similar or greater neural activation compared to the other conditions (Fig. 10).

The experimental results also emphasize the importance of motor intention during the therapy. The assisted movement is synchronized to the motor intention in RMT condition since the movement of the unaffected hand induces the movement of the affected hand. In RT condition, the affected hand is moved passively without intention. Even though the same sensory feedback is induced for the affected hand including visual and somatosensory feedback in both conditions, the activation on the contralateral motor cortex was larger in the RMT condition (Fig. 9). This result indicates that synchronization of the sensory feedback and motor intention is important for enhancing the neural activation in the motor cortex.

Individuals with chronic stroke have been shown to experience interhemispheric imbalance, likely caused by failure to release interhemispheric inhibition from the intact to the damaged hemisphere before movement execution [37, 38]. Patients with successful motor rehabilitation tend to show improvements in interhemispheric balance while patients with poor motor recovery do not show significant changes [38, 39]. Therefore, it is important to induce balanced interhemispheric brain activation for actual functional recovery of the affected hand. As shown in the cortical activation results in MT condition the contralateral motor cortex of the affected hand shows significantly smaller activation compared to the ipsilateral motor cortex (Fig. 11), which could intensify interhemispheric imbalance. On the other hand, cortical activation results in RMT condition showed balanced activation of the contralateral and ipsilateral motor cortex (Fig. 11). We hypothesize that the balanced cortical activation observed in RMT will enhance neural recovery compared to the less balanced activation observed in RT and MT.

This study is limited to a cross-sectional study observing the neural effect of RMT. A long-term study should be further conducted to compare the neural changes and functional improvements by the enlarged and hemispherically balanced neural stimulation on the contralateral motor cortex through RMT.

## Conclusions

The proposed hand rehabilitation system allows the user to train the affected hand with the actuated soft robotic glove by using the movement of the unaffected limb measured by the sensor glove. RMT provided with the proposed rehabilitation system enabled an increase of the neural activity of the contralateral motor cortex and induce balanced interhemispheric cortical activity during grasping. This study shows the importance of considering cortical activation when designing training protocols with rehabilitation robots. While RT inducing passive movements has shown similar or smaller functional recovery compared to conventional therapy conducted by occupational therapists [40, 41], RMT involving the intention of the user in the therapy may enhance functional recovery as larger neural stimulation could be provided on the contralateral motor cortex (Fig. 8) of the affected hand. RMT by using the proposed hand rehabilitation system can be applied for self-rehabilitation at home allowing intensive functional training that effectively promotes neuroplastic changes through enlarged neural activation of the motor cortex.

## Data Availability

The datasets used and/or analyzed during the current study are available from the corresponding author on reasonable request.
